# Strategy for successful expression of the *Pseudomonas putida* nitrile hydratase activator P14K in *Escherichia coli*

**DOI:** 10.1186/1472-6750-13-48

**Published:** 2013-06-03

**Authors:** Yi Liu, Wenjing Cui, Yueqin Fang, Yuechun Yu, Youtian Cui, Yuanyuan Xia, Michihiko Kobayashi, Zhemin Zhou

**Affiliations:** 1 Key Laboratory of Industrial Biotechnology, Ministry of Education, School of Biotechnology, Jiangnan University, Wuxi, 214122, Peoples Republic of China; 2 School of Environmental and Civil Engineering, Jiangnan University, Wuxi, 214122, Peoples Republic of China; 3 Institute of Applied Biochemistry, and Graduate School of Life and Environmental Sciences, The University of Tsukuba, 1-1-1 Tennodai, Tsukuba, Ibaraki, 305-8572, Japan

**Keywords:** NHase, N-end rule, *Pseudomonas putida*, P14K, Stability

## Abstract

**Background:**

Activators of Nitrile hydratase (NHase) are essential for functional NHase biosynthesis. However, the activator P14K in *P. putida* is difficult to heterogeneously express, which retards the clarification of the mechanism of P14K involved in the maturation of NHase. Although a strep tag containing P14K (strep-P14K) was over-expressed, its low expression level and low stability affect the further analysis.

**Results:**

We successfully expressed P14K through genetic modifications according to N-end rule and analyzed the mechanism for its difficult expression. We found that mutation of the second N-terminal amino-acid of the protein from lysine to alanine or truncating the N-terminal 16 amino-acid sequence resulted in successful expression of P14K. Moreover, fusion of a pelB leader and strep tag together (pelB-strep-P14K) at the N-terminus increased P14K expression. In addition, the pelB-strep-P14K was more stable than the strep-P14K.

**Conclusions:**

Our results are not only useful for clarification of the role of P14K involved in the NHase maturation, but also helpful for heterologous expression of other difficult expression proteins.

## Background

Nitrile hydratase (NHase, EC 4.2.1.84) is composed of α- and β-subunits. The enzyme contains either a non-heme iron (Fe-NHase) [1] or non-corrin cobalt ion (Co-NHase) [2] in the active center and catalyzes the hydration of a nitrile to the corresponding amide, which is followed by several consecutive reactions: amide → acid → acyl-CoA, as catalyzed by amidase [3] and acyl-CoA synthetase [1], respectively. The metal ions in both Co-NHase and Fe-NHase are located in their α-subunits, which share a characteristic metal-binding motif [CXLC(SO_2_H)SC(SOH)] containing two modified cysteine residues: cysteine-sulfinic acid (αCys-SO_2_H) and cysteine-sulfenic acid (αCys-SOH) [1,4,5]. The apoenzyme is likely to be unmodified, according to previous studies on NHase [6] and a related enzyme, thiocyanate hydrolase (SCNase) [7].

The trafficking of metal ions into NHases is mediated by various “activator proteins” [8]. Fe-NHases require activators for functional expression in *Rhodococcus* sp. N-771 [9], *Pseudomonas chlororaphis* B23 [10] and *Rhodococcus* sp. N-774 [11]. A proposed metal-binding motif, CXCC, in the NHase activator of *Rhodococcus* sp. N-771 has been identified and the activators for Fe-type NHases have been shown to act as metallochaperones [12]. For the two Co-NHases (L-NHase and H-NHase) in *Rhodococcus rhodochrous* J1, cobalt incorporation has been found to be dependent on self-subunit swapping: the activator protein exists as a complex with the α-subunit of NHase, the cobalt incorporation involves the swapping of the cobalt-free α-subunit of the cobalt-free NHase with the cobalt-containing α-subunit of the complex [13-15]. NHase in *Pseudomonas putida* NRRL-18668 and acetonitrile hydratase (ANHase, an NHase that catalyzes the hydration of small aliphatic nitriles) from *Rhodococcus jostii* RHA1 are also Co-NHases, in which P14K and AnhE, respectively, are essential for NHase maturation [16,17]. However, their gene organizations are quite different from those of L-NHase and H-NHase. The structural genes of L-NHase and H-NHase have the order < β-subunit > <α-subunit > <self-subunit swapping chaperone>, while those in ANHase and the NHase of *P. putida* NRRL-18668 have the order < α-subunit > <AnhE > <β-subunit > [16] and < α-subunit > <β-subunit > <P14K > [17], respectively, with the latter protein being identical to the metallochaperone in Fe-NHase except that the molecular mass of the protein in Fe-NHase is larger than P14K. While AnhE has been found to act as a metallochaperone (not as a self-subunit swapping chaperone) during cobalt incorporation into ANHase [16], very recently, we discovered that cobalt incorporation into the NHase of *P. putida* NRRL-18668 is also dependent on the self-subunit swapping, and the P14K is a complex with the α-subunit [18]. However, the P14K is difficult to be heterogeneously expressed, though a strep tag containing P14K was expressed, its low expression level and low stability retard the further clarification of their detailed role for cobalt incorporation.

**Figure 1:**
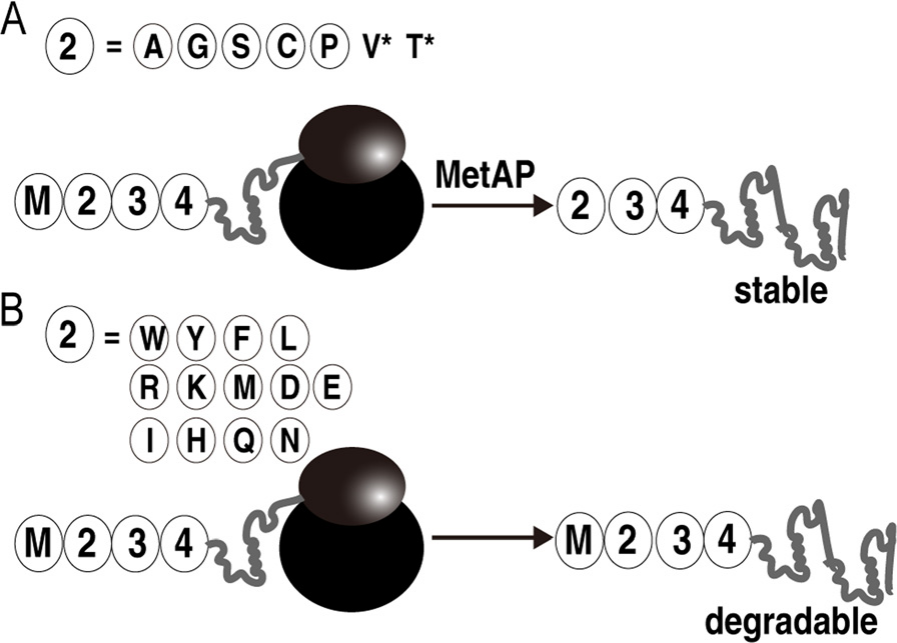
**The schematic diagram of N-end rule in bacteria.** (**A**) Relatively long protein half-life. If the second N-terminal amino-acid is A, G, S, C or P and in some cases V or T, which can be processed by methionine aminopeptidase (MetAP), the protein will have a long half-life. (**B**) Relatively short protein half-life. When the second N-terminal amino-acid is W, Y, F, L, R, K, M, D, E, I, H, Q or N, which can not be processed by MetAP, the protein will have a short half-life.

Heterologous expression systems are commonly used for protein research. Protein degradation in heterologous expression systems often leads to failure for the isolation of proteins of interest. Intracellular protein degradation plays an essential role in many physiological processes by removing damaged polypeptides and proteins that harbor specific destruction tags. N-end pathway degradation relates the metabolic stability of a protein to the N-terminal residue of that protein [19]. The N-end rule defines the stability of proteins according to the nature of their N-terminal residues. These residues are classified as stabilizing and destabilizing residues, which serve as recognition determinants for protein degradation [19-21]. In bacteria such as *E. coli*, the N-end rule pathway is present. According to the N-end rule, amino-terminal arginine, lysine, leucine, phenylalanine, tyrosine and tryptophan confer 2 minute half-lives to proteins, while the other amino-terminal residues confer greater than 10 hour half-lives to the same proteins (Figure 1) [22,23].

In the present study, we successfully expressed the NHase activator P14K through site-specific mutagenesis taking into account N-end rule degradation. We also increased the expression and the stability of P14K by fusion of a pelB leader and strep tag together at its N-terminus. These results are useful for elucidation of the mechanism of cobalt incorporation into the α-subunit of NHase in *P. putida* NRRL-18668. Furthermore, these strategies, which promote the over-expression of the instable P14K in *E. coli*, might also be helpful for the heterologous expression of other difficult expression proteins.

## Results and discussion

### Molecular modification to improve the yield of P14K from *P. putida* NRRL-18668

**Figure 2:**
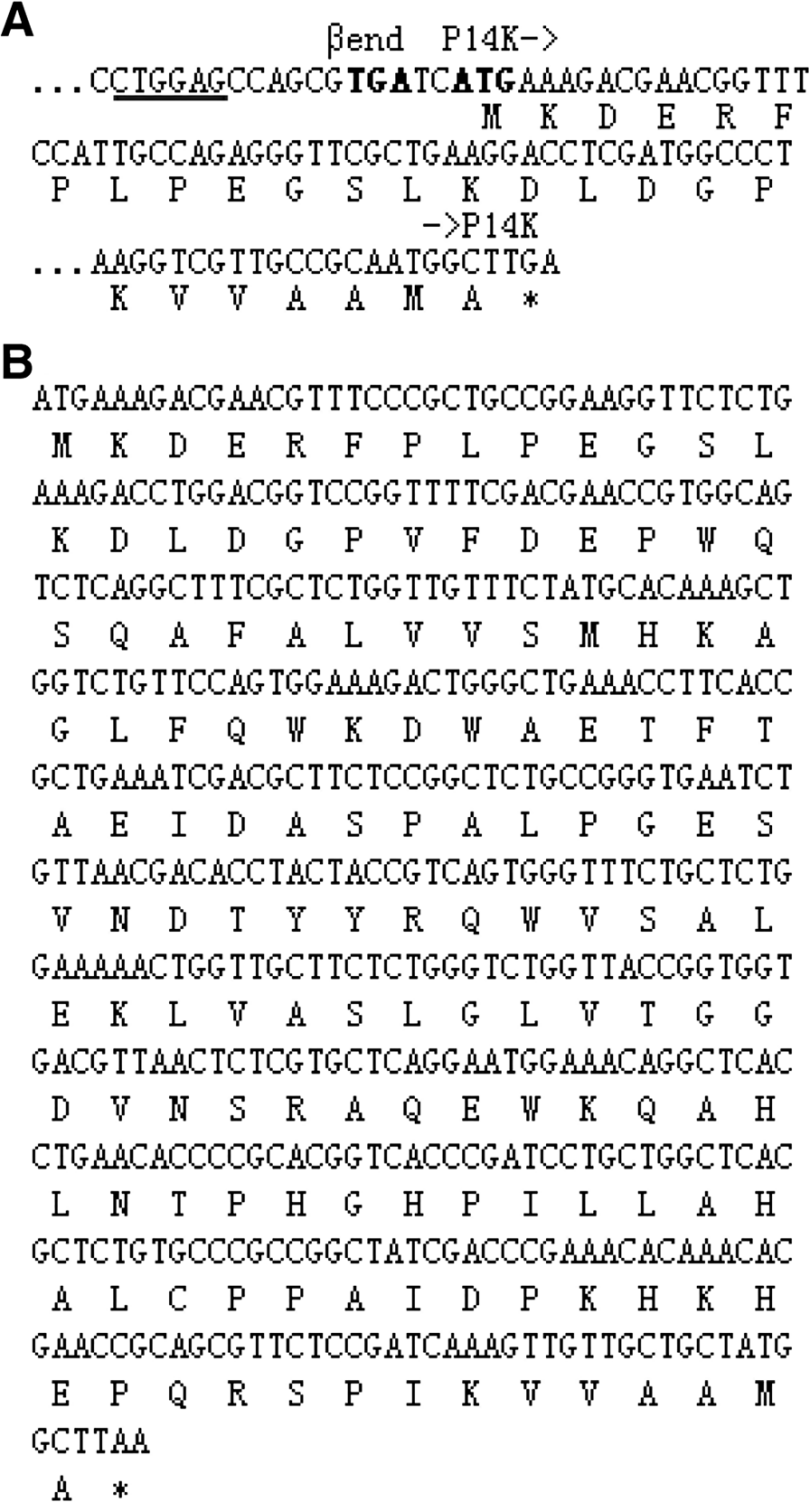
**Potential ribosome-binding site of P14K and the codon-optimized nucleotide sequence of P14K.** (**A**) Nucleotide sequence of P14K. The underlined nucleotide sequence denotes the potential ribosome-binding site. (**B**) Nucleotide sequence of the codon-optimized P14K (*Po*) nucleotide sequence.

**Figure 3:**
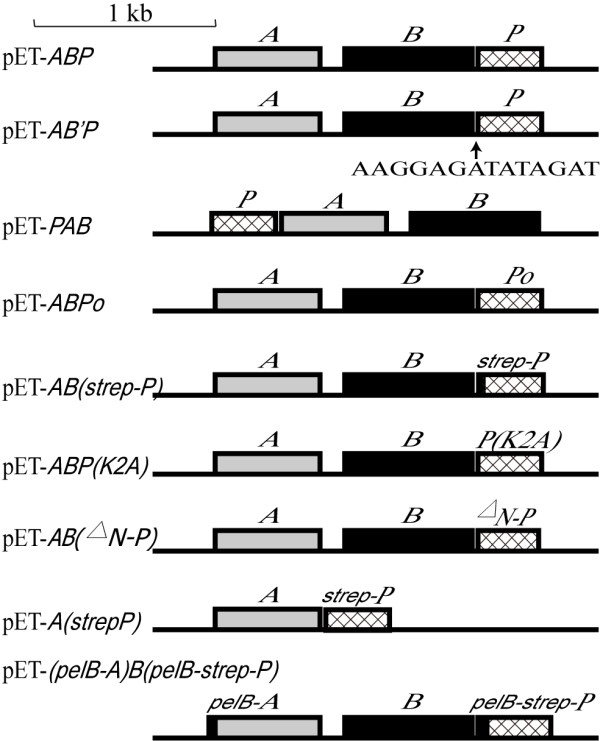
**Sketch of the plasmids used in this study.** pET-*ABP*, a plasmid containing the *ABP* genes; pET-*AB’P*, a plasmid containing the *ABP* genes with an enhanced RBS (AAGGAG) between the *B* and *P* genes; pET-*PAB*, a plasmid containing the *ABP* genes with a < *P* > <*A* > <*B* > order; pET-*ABPo*, a plasmid containing the *AB* and the codon-optimized *P* genes; pET-*AB(strep-P)*, a plasmid containing the *AB* and a strep-tagged *P* genes [18]; pET-*ABP(K2A)*, a plasmid harboring the *AB* and a mutant *P* (the second lysine (K) is mutated to alanine (A)) genes; pET-*AB(*^*△*^*N-P)*, a plasmid harboring the *AB* and a 16 amino-acid truncated N terminus *P* genes; pET-*A(strep-P)*, a plasmid containing the *A* and a strep-tagged *P* genes; pET-*(pelB-A)B(pelB-strep-P)*, a pET-22b plasmid containing the *pelB-A*, *B* and *pelB-strep-P* genes.

**Figure 4:**
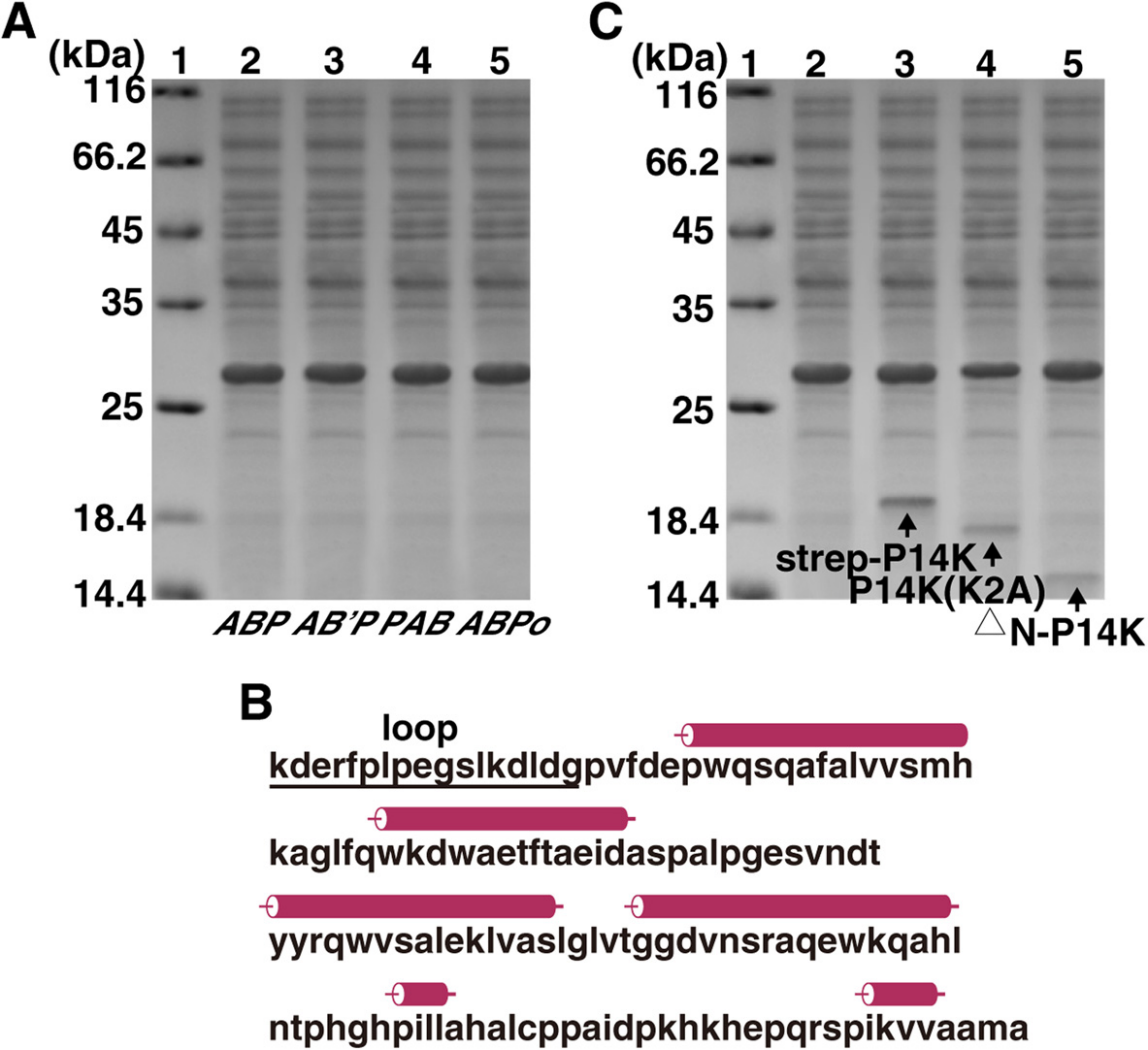
**SDS-PAGE analysis of recombinant NHases and P14K, and the predicted secondary structure of P14K.** (**A**) SDS-PAGE analysis of the cell extracts of the transformants carrying pET-*ABP*, pET-*AB’P*, pET-*PAB* or pET-*ABP*o. 1, mark; 2, pET-*ABP*; 3, pET-*AB’P*; 4, pET-*PAB*; 5, pET-*ABPo*. (**B**) Predicted secondary structure of P14K. (**C**) SDS-PAGE analysis of the cell extracts of the transformants carrying pET-*AB(strep-P)*, pET-*ABP(K2A)* or pET-*AB(*^*△*^*N-P)*. 1, mark; 2, pET-*ABP*; 3, pET-*AB(strep-P)*; 4, pET-*ABP(K2A)*; 5, harboring pET-*AB(*^*△*^*N-P)*.

To clarify the mechanism of cobalt incorporation into the NHase of *P. putida* NRRL-18668, we attempted to isolate P14K, which is essential for NHase activity [17]. The transformant harboring pET-*ABP,* which contained the NHase and P14K genes, was used for NHase and P14K expression. As a result, although NHase was successfully expressed, P14K was hardly detected on SDS-PAGE. This result is consistent with the previous report [17]. Although a strep tag containing P14K was expressed using a transformant harboring pET-*AB(strep-P)*, and the strep-P14K was found to be a complex with the α-subunit [α-(strep-P14K)] [18], the low expression level and the low stability of the complex retard the further clarification of its detailed role for cobalt incorporation, a strategy for enhancing P14K expression level and stability is expected. It has been shown that heterologous expression of proteins in *E. coli* can be enhanced by using a synthetically strong ribosome binding site (RBS) [24], reducing the distance between the promoter region and target gene [25] and gene codon optimization [26]. To enhance the expression of P14K, the putative RBS (CTGGAG) within the *B* gene (encoding the β subunit) (Figure 2A) was replaced with an enhanced RBS (AAGGAG) between gene *B* and gene *P* (encoding P14K) during the construction of the plasmid pET-*AB’P* (Figure 3). Simultaneously, the plasmid pET-*PAB*, with the gene order < *P* > <*A* > <*B*>, was constructed. In this plasmid, the distance between the promoter sequence and the P14K gene was shortened and the strong RBS was inserted upstream of each gene (Figure 3). Moreover, plasmid pET-*ABPo,* with optimized P14K gene codons and a strong RBS for each gene, was also constructed (Figure 2B). However, P14K was not abundantly expressed by the transformants harboring pET-*AB’P*, pET-*PAB* or pET-*ABPo* (Figure 4A).

### Successful heterologous expression of P14K

The N-end rule states that the half-life of a protein is determined by the nature of its N-terminal residue [19-21]. This fundamental principle of proteolytic regulation is conserved from bacteria to mammals [19]. N-terminal arginine, lysine, leucine, phenylalanine, tyrosine and tryptophan confer 2 minute half-lives to proteins, while the other N-terminal residues confer greater than 10 hour half-lives to the same proteins [22,23] (Figure 1). We analyzed the N-terminal amino-acids of some weakly expressed NHase activators from *Bordetella petrii* DSM 12804, *P. putida* NRRL-18668, *Bacillus* BR449 and *Bacillus* RAPc8. Surprisingly, we found that all of the second N-terminal amino-acids of the NHase activators in these four strains are lysine (K) (Table 1). Thus, it may be that N-end rule degradation leads to difficulty with the expression and isolation of P14K. Based on this speculation, we designed a mutant gene, *ABP(K2A)*, in which the Lys-2 in the N-terminus of P14K was substituted with Ala in the plasmid pET-*ABP(K2A)*. In addition, we analyzed the secondary structure of the P14K as predicted by JPRED3 (http://www.compbio.dundee.ac.uk/www-jpred/index.html) and found the N-terminal 16 amino-acid group is in an N-terminal loop (Figure 4B) and the 17th amino-acid is a Gly that could avoid P14K degradation effectively according the N-end rule [22,23]. Therefore, we constructed plasmid pET-*AB(*^*△*^*N-P)*, in which the N-terminal 16 residues of P14K were deleted, the second N-terminal amino-acid in the truncated P14K was Gly. The transformants harboring pET-*ABP(K2A)* and pET-*AB(*^*△*^*N-P)* were used for NHase and P14K expression. As shown in Figure 4C, each mutant P14K was successfully expressed and the crude NHase activity in the cell-free extracts of each transformant was similar to that of the transformant harboring pET-*ABP* (120.5 U/mg). However, compared with the expression of P14K using the transformant harboring pET-*AB(strep-P)*, the expression level of P14K in the two mutants was not improved (Figure 4C).

**Table 1:** N-terminal amino-acid sequence of NHase activators in various strains

**Strain**	**N-terminal amino-acid sequence**
*Bordetella petrii* DSM 12804	M K DERLPLP (YP_001630019.1)
*Pseudomonas putida* NRRL-18668	M K DERFPLP (P14K in this study*)
*Bacillus* BR449	M K SCENQPN (AAF69003.1)
*Bacillus* RAPc8	M K SCENQPN (AAS84452.1)

Protein accession numbers are shown in brackets. * the correct N-terminal amino-acid sequence of P14K was identified by our previous work [18].

### High P14K expression yield

**Figure 5:**
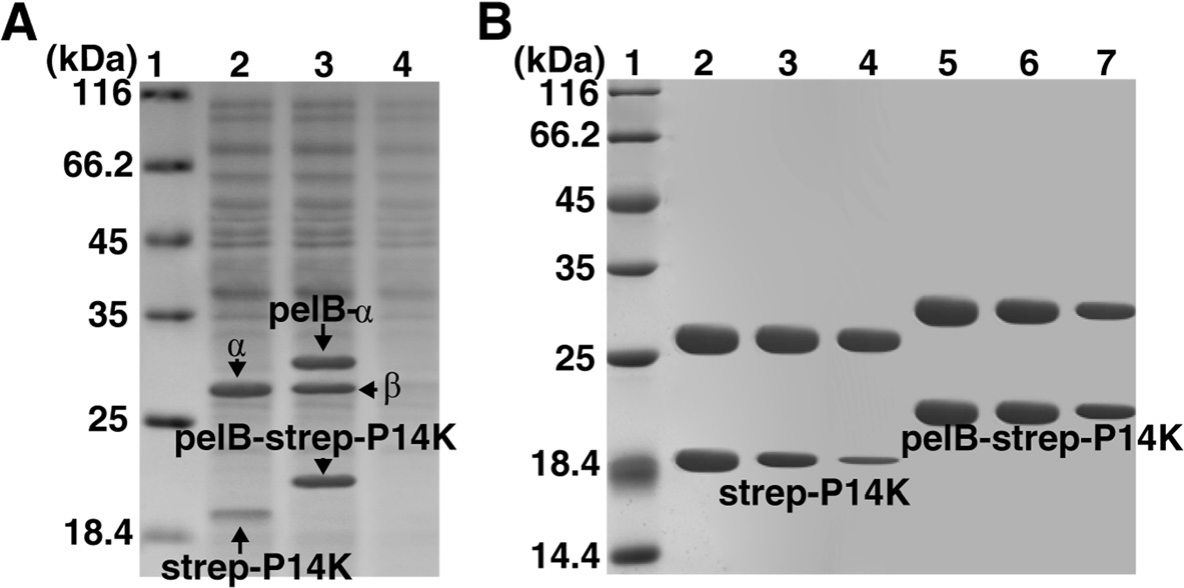
**SDS-PAGE analysis of recombinant pelB-strep-P14K. (A)** SDS-PAGE analysis of the activators encoded by *A(strep-P)* and (*pelB-A)B(pelB-strep-P)*. 1, mark; 2, cell extracts of the transformant containing pET-*A(strep-P)*; 3, cell extracts of the transformant containing pET-(*pelB-A)B(pelB-strep-P)*; 4, culture supernatant of the transformant containing pET-(*pelB-A)B(pelB-strep-P)*. (**B**) SDS-PAGE analysis of stability of purified activator complexes [α-(strep-P14K)] and [α-(pelB-strep-P14K)]. 1, mark; 2, purified [α-(strep-P14K)]; 3, purified [α-(strep-P14K)] after storage for 1 day; 4, purified [α-(strep-P14K)] after storage for 2 days; 5, purified [α-(pelB-strep-P14K)]; 6, purified [α-(pelB-strep-P14K)] after storage for 2 days; 7, purified [α-(pelB-strep-P14K)] after storage for 6 days.

It has been reported that exporting a protein to the periplasm to enhance its stability can be regarded as an effective strategy to optimize the production of recombinant proteins [27,28]. To yield large amounts of P14K in recombinant *E. coli* cells, we attempted to secrete P14K into the periplasmic space. As P14K formed a complex with the α-subunit of NHase [18], we designed a mutant gene (*pelB-A)B(pelB-strep-P)* in which the pelB signal peptide was added upstream of the *A* and *strep-P* genes in the plasmid pET22b-(*pelB-A)B(pelB-strep-P)*. The transformant harboring pET22b-(*pelB-A)B(pelB-strep-P)* was used for recombinant P14K expression. Although no target protein was observed in the culture supernatant, a large amount of the full-length pelB-strep-P14K and pelB-α subunit (the pelB signal peptides were not cut off in either) were detected in the cell-free extract (Figure 5A). In addition, the enzyme activity in the cell-free extract was comparable to that of the wild-type NHase.

### Stability of pelB-strep-P14K

To investigate the mechanism of how the fusion protein pelB-strep enhances the recombinant expression of P14K, we compared the difference in the protein stability between the purified P14K-containing activator complex [α-(strep-P14K)] and [α-(pelB-strep-P14K)]. SDS-PAGE analysis was carried to investigate the stability of cobalt-containing [α-(strep-P14K)] and [α-(pelB-strep-P14K)] (a culture containing the cobalt ion) during storage at room temperature. As shown in Figure 5B, the pelB-strep-P14K band from the recombinant [α-(pelB-strep-P14K)] complex decayed to 90% of the original intensity after 2 days of storage and eventually to 60% after 6 days. However, the strep-P14K band from the [α-(strep-P14K)] complex decreased to 60% after only 1 day of storage and to 10% after 2 days. The finding that pelB-strep-P14K is far more stable than strep-P14K indicated that thermal stability may be a key factor in P14K expression.

## Conclusions

In conclusion, the activator P14K from *P. putida* NRRL-18668 was successfully expressed based on the N-end rule degradation, the stability of the P14K was improved by adding a pelB signal peptide. Further study of the influence of P14K on the maturation of NHase is currently underway. The strategy used for P14K expression in this study may be useful for the heterologous expression of other difficult expression proteins.

## Methods

### Bacterial strain and plasmids

NHase and the P14K gene (*ABP)* were cloned from *P. putida* NRRL-18668. *E. coli* BL21 (DE3) was used as the host for the plasmid pET-24a(+), which was used for *ABP*, *AB’P*, *PAB*, *ABPo*, *AB(strep-P)*, *ABP(K2A)*, *AB(*^*△*^*N-P)* and *A(strep-P)* expression. The plasmid pET-22b was used for (*pelB-A)B(pelB-strep-P)* expression.

### Construction of plasmids

The genomic DNA of *P. putida* NRRL-18668 was isolated for *ABP* cloning with the primers A-up and P-down (Table 2 and Figure 3). The PCR products were digested and ligated into pET-24a(+) to generate the plasmid pET-*ABP*. The recombinant plasmid pET-*ABP* was then transformed into *E. coli* BL21 (DE3) for heterologous expression. An overlap extension PCR protocol was used for construction of the plasmid pET-*AB’P* (*’*represents an insertion of RBS sequence) through two rounds of PCR. The first round of PCR was performed using the primer pairs A-up and B-down(rbs) and P-up(rbs) and P-down, respectively, the plasmid pET-*ABP* was used as the template. The second round of PCR was performed to produce the full-length *AB’P,* using the primers A-up and P-down and mixing equal molar amounts of the first-round products as template. The following plasmids were prepared similar to pET-*AB’P*. The plasmid pET-*PAB* was prepared with the primer pairs P-up and P-down(PAB) and A-up(PAB) and B-down(PAB). The plasmid pET-*ABPo* was constructed with the primer pairs A-up and B-down(rbs) and Po-up and Po-down. The plasmid pET-*Po* (*Po*, codon optimized *P* gene synthesized by Sangon Biotech Ltd.) was used as the template. The plasmid pET-*AB(strep-P)* was constructed with the primer pairs A-up and B-down(strep) and P-up(strep) and P-down as described previously [18]. The plasmid pET-*AB(*^*△*^*N-P)* was constructed with the primers A-up and B-down(rbs) and P-up(^△^N) and P-down. The plasmid pET-*A(strep-P)* was generated from pET-*ABP* with the primers A-up and A-down(strep) and P-up(strep) and P-down. The plasmid pET22b-(*pelB-A)B(pelB-strep-P)* was prepared with the primers of secA-up and secB-down and secP-up and P-down. The plasmid pET-*ABP(K2A)* mutant was constructed to produce full-length *ABP(K2A)* with the primers K2A-up and K2A-down using pET-*AB’P* as the template. The PCR products were digested with DpnI to degrade the template plasmid and then transformed into *E. coli* BL21 (DE3).

**Table 2:** Oligonucleotide primers used in this study

**Name**	**Sequence**
A-up	5’-GGAATTC*CATATG*GGGCAATCACACACGC-3’
P-down	5’-CCG*GAATTC*TCAAGCCATTGCGGCAACGA-3’
B-down(rbs)	5’-ATATCTATATCTCCTTTCACGCTGGCTCCAGGTAGTC-3’
P-up(rbs)	5’-TGAAAGGAGATATAGATATGAAAGACGAACGGTTT-3’
P-up	5’-GGAATTC*CATATG*AAAGACGAACGGTTT-3’
P-down(PAB)	5’-CATATCTATATCTCCTTTCAAGCCATTGCGGCAACG-3’
A-up(PAB)	5’-TGAAAGGAGATATAGATATGGGGCAATCACACACGC-3’
B-down(PAB)	5’-CCG*GAATTC*TCACGCTGGCTCCAGGTAGT-3’
Po-up	5’-GGAATTC*CATATG*AAAGACGAACGTTTC-3’
Po-down	5’-CCG*GAATTC*TCAAGCCATAGCAGCAACAA-3’
B-down(strep)	5’-GCGGGTGGCTCCAGCTTGCCATATCTATATCTCCTTTCACGCTGGCTCCAGGTAGTCATC-3’
P-up(strep)	5’-AAGCTGGAGCCACCCGCAGTTCGAAAAGGGTGCAAAAGACGAACGGTTTCCATT-3’
K2A-up	5’-TGAAAGGAGATATAGATATGGCGGACGAACGGTTTC-3’
K2A-down	5’-GCAATGGAAACCGTTCGTCCGCCATATCTATATCTCC-3’
P-up(^△^N)	5’-TGAAAGGAGATATAGATATGGGCCCTGTGTTTGACG-3’
A-down(strep)	5’-GCGGGTGGCTCCAGCTTGCCATATCTATATCTCCTTTTAATGAGATGGGGTGGGTTGGGT-3’
secA-up	5’-CCG*GAATTC*GGGGCAATCACACACGCAT-3’
secB-down	5’-GATATCCATGGCCATCGCCGGCTGGGCAGCGAGGAGCAGCAGACCAGCAGCAGCGGTCGGCAGCAGGTATTTCATATGTATATCTCCTTTCACGCTGGC-3’
secP-up	5’-CAGCCGGCGATGGCCATGGATATCGGAATTAATGCAAGCTGGAGCCACCCGCAGTTCGAAAAGGGTGCAAAAGACGAACGGTTTCCATTGCCAG-3’
secP-down	5’-CGACCC*AAGCTT*TCAAGCCATTGCGGCAACGACC-3’

Italicized letters denote the NdeI, EcoRI and HindIII recognition sites, respectively.

### Expression and purification of enzymes and enzyme assay

*E. coli* BL21 (DE3) transformants containing the recombinant plasmids were grown at 37°C in TB medium containing CoCl_2_.6H_2_O (0.05 g/l) and kanamycin (50 μg/ml) until culture *A*_600_ reached 0.8. Isopropyl β-D-thiogalactopyranoside was added to a final concentration of 0.4 mM. The cells were then incubated at 24°C for 16 h.

All purification steps were performed at 4°C. The procedures were conducted with an AKTA purifier (GE Healthcare UK Ltd.). Potassium phosphate buffer (KPB) (10 mM, pH 7.5) containing 0.5 mM dithiothreitol (DTT) was used in the purification steps. Both NHase and its activator complex were purified with a HisTrap HP column (GE Healthcare UK Ltd.). The target proteins were eluted off the column with gradient concentrations of imidazole from 0 mM to 500 mM (40 mM, 80 mM, 200 mM, 300 mM and 500 mM) in 10 mM KPB. The preliminarily separated proteins were further purified with a Hiload 16/60 Superdex 200 pg column (GE Healthcare UK Ltd.). The process of separation and purification was monitored by SDS-PAGE analysis.

NHase activity was assayed in a reaction mixture comprising 10 mM KPB (pH 7.5), 20 mM 3-cyanopyridine as a substrate and 0.1 μg enzyme in a total volume of 500 μL. The reaction mixture was incubated at 20°C for 20 min and terminated by addition of 500 μL of acetonitrile. The activity of NHase was determined by monitoring the formation of nicotinamide in the reaction mixture with high-pressure liquid chromatography (HPLC) as previously described [13]. One unit of NHase activity was defined as the amount of enzyme that catalyzed the formation of 1 μmol of nicotinamide per min at 20°C.

## Competing interests

The authors declare that there are no competing interests.

## Authors’ contributions

ZMZ, WJC and MK designed this study. YL, YQF, YCY, YTC and YYX performed the experimental work. ZMZ, WJC, YL and MK wrote the manuscript. ZMZ and MK collaborated in the coordination of the research and helped to draft the manuscript. All authors read and approved the submission of the manuscript.
